# Group problem management plus (PM+) to decrease psychological distress among Syrian refugees in Turkey: a pilot randomised controlled trial

**DOI:** 10.1186/s12888-021-03645-w

**Published:** 2022-01-04

**Authors:** C. Acarturk, E. Uygun, Z. Ilkkursun, T. Yurtbakan, G. Kurt, J. Adam-Troian, I. Senay, R. Bryant, P. Cuijpers, N. Kiselev, D. McDaid, N. Morina, Z. Nisanci, A. L. Park, M. Sijbrandij, P. Ventevogel, D. C. Fuhr

**Affiliations:** 1 Department of Psychology, Koc University, Istanbul, Turkey; 2 Trauma and Disaster, Mental Health, Bilgi University, Istanbul, Turkey; 3 Department of Psychology, Istanbul Medipol University, Istanbul, Turkey; 4 Department of International Studies, American University of Sharjah, Sharjah, United Arab Emirates; 5 Department of Psychology, Istanbul Sehir University, Istanbul, Turkey; 6 School of Psychology, University of New South Wales, Sydney, Australia; 7 Department of Clinical, Neuro and Developmental Psychology and WHO Collaborating Centre for Research and Dissemination of Psychological Interventions, Amsterdam Public Health research institute, Vrije Universiteit Amsterdam, Amsterdam, Netherlands; 8 Department of Consultation-Liaison Psychiatry and Psychosomatic Medicine, University Hospital Zurich, University of Zurich, Zurich, Switzerland; 9 Department of Health Policy, Care Policy and Evaluation Centre, London School of Economics and Political Science, London, UK; 10 Department of Applied Sociology, Marmara University, Istanbul, Turkey; 11 United Nations High Commissioner for Refugees, Public Health Section, Genève, Switzerland; 12 Department of Health Services Research and Policy, London School of Hygiene and Tropical Medicine, London, UK

**Keywords:** Refugees, Common mental health problems, Group intervention, Task sharing, Pilot, Randomised controlled trial, Feasibility

## Abstract

**Background:**

Syrian refugees resettled in Turkey show a high prevalence of symptoms of mental disorders. Problem Management Plus (PM+) is an effective psychological intervention delivered by non-specialist health care providers which has shown to decrease psychological distress among people exposed to adversity. In this single-blind pilot randomised controlled trial, we examined the methodological trial procedures of Group PM+ (gPM+) among Syrian refugees with psychological distress in Istanbul, Turkey, and assessed feasibility, acceptability, perceived impact and the potential cost-effectiveness of the intervention.

**Methods:**

Refugees with psychological distress (Kessler Psychological Distress Scale, K10 > 15) and impaired psychosocial functioning (World Health Organization Disability Assessment Schedule, WHODAS 2.0 > 16) were recruited from the community and randomised to either gPM+ and enhanced care as usual (E-CAU) (*n* = 24) or E-CAU only (*n* = 22). gPM+ comprised of five weekly group sessions with eight to ten participants per group. Acceptability and feasibility of the intervention were assessed through semi-structured interviews. The primary outcome at 3-month follow-up was symptoms of depression and anxiety (Hopkins Symptoms Checklist-25). Psychosocial functioning (WHODAS 2.0), symptoms of posttraumatic stress disorder and self-identified problems (Psychological Outcomes Profiles, PSYCHLOPS) were included as secondary outcomes. A modified version of the Client Service Receipt Inventory was used to document changes in the costs of health service utilisation as well as productivity losses.

**Results:**

There were no barriers experienced in recruiting study participants and in randomising them into the respective study arms. Retention in gPM+ was high (75%). Qualitative analyses of the interviews with the participants showed that Syrian refugees had a positive view on the content, implementation and format of gPM+. No adverse events were reported during the implementation. The study was not powered to detect an effect. No significant difference between gPM+ and E-CAU group on primary and secondary outcome measures, or in economic impacts were found.

**Conclusions:**

gPM+ delivered by non-specialist peer providers seemed to be an acceptable, feasible and safe intervention for Syrian refugees in Turkey with elevated levels of psychological distress. This pilot RCT sets the stage for a fully powered RCT.

**Trial registration:**

ClinicalTrials.gov Identifier NCT03567083; date: 25/06/2018.

**Supplementary Information:**

The online version contains supplementary material available at 10.1186/s12888-021-03645-w.

## Introduction

The number of forcibly displaced people is at its highest with more people being currently displaced than after the second world war [1]. A large proportion of forcibly displaced persons seek refuge, commonly in neighbouring countries [1]. 6.7 million Syrian refugees have fled their home country and sought refuge as a result of the civil war. Currently, Turkey hosts the largest number of refugees and asylum seekers worldwide which amounts to 4 million [2] including 3.6 million Syrian refugees [3].

Recent research indicates that Syrian refugees are exposed to potentially traumatic events including life threats, torture, loss of loved ones in Syria and during the flight [4–7]. A growing number of studies have also shown that Syrian refugees experience a wide range of post-displacement problems such as language problems, discrimination [8–10], and economic problems after they arrive in recipient countries [6, 7, 11]. This may worsen existing mental health problems and new symptoms of depression, anxiety and posttraumatic stress disorder (PTSD) may arise [6, 10, 11]. Reports also indicate an increase in severe mental health problems and suicidality among Syrian refugees [12]. Moreover, mental health symptoms among refugees are associated with loss in quality of life [14] and impaired psychosocial functioning [15–18]. However, despite the high burden of common mental disorders, access to mental health care among Syrian refugees in Turkey is low [13] with only around 8% of Syrian refugees with mental disorders receiving treatment [13]. This is because of various reasons including language problems, stigma and lack of culturally adapted psychosocial interventions which address Syrian refugee’s specific needs.

Psychosocial interventions are effective in decreasing symptoms of post-traumatic stress disorder (PTSD), depression, and anxiety among refugees [19]. However, most of these interventions are provided by specialised mental health care providers who may need the assistance of trained interpreters during the delivery of the intervention. The number of interpreters who can help with intervention delivery are lacking in Turkey, therefore, [20] this approach is not feasible or scalable [21]. Brief, peer to peer, transdiagnostic interventions may be more appropriate [22] and may facilitate access to care by implementing a culturally adapted psychological intervention in the community.

To reduce mental and psychosocial problems among people living in adversity, and to take account of the high comorbidity between mental health problems [7, 23], the World Health Organization (WHO) has developed a 5-session psychosocial intervention called Problem Management Plus (PM+) [24]. This is a brief, scalable intervention that can be delivered by trained and supervised non-specialist peer providers. It includes four evidence-based strategies (a) stress management, (b) problem solving, (c) behavioural activation, and (d) accessing social support. Studies in Peshawar (Pakistan) and Nairobi (Kenya) indicated that the individual version of PM+ is effective in decreasing psychological distress and leads to improved psychosocial functioning [25–27]. A group version of PM+ has been developed as well (gPM+) [28] which has been found to be effective in reducing symptoms of anxiety and depression among women living in a post-conflict setting in Pakistan [29].

Given the large number of Syrian refugees in Turkey, their mental health needs and the inadequacy of the current health system to respond properly to their psychosocial needs, we adapted gPM+ for Syrian refugees in Turkey as a part of the EU-funded STRENGTHS project which intends to scale up PM+ for Syrian refugees in countries neighbouring Syria and European countries [30]. The current study presents findings from a pilot randomised controlled trial (RCT) to examine (1) the methodological procedures of randomization, recruitment, data collection and retention prior to conducting a fully-powered RCT, and (2) to investigate feasibility, acceptability and perceived impact of the adapted gPM+ version to reduce psychological distress among Syrian refugees in Turkey. Although not powered to show an effect we also explored potential treatment effects as well as impacts on resource utilisation and costs. The latter would be needed for a future economic evaluation.

## Methods

This study was a two-arm, pilot randomised control trial (RCT) in which the outcome assessors were blinded to the study condition of the participants. The trial was approved by the Research Ethics Committee of Istanbul Sehir University (Protocol ID:12/2017) and prospectively registered online (NCT03567083). This study was conducted according to the principles of the Declaration of Helsinki Decleration, and adhered to the International Conference on Harmonisation (ICH), the WHO Good Clinical Practice standards (GCP), and the Medical Research Involving Human Subjects Act (WMO). Informed consent for participating in the study was received from all participants.

### Setting and study population

The project was conducted in collaboration with the Refugee and Asylum Seekers Assistance and Solidarity Association (RASASA) in Turkey, a non-governmental organisation (NGO) which provides health, psychosocial and legal support to Syrians in need. The study was implemented in Sultanbeyli, a suburb of Istanbul which hosts more than 30,000 Syrian refugees. Participants were recruited via dissemination of brochures and posters in RASASA to its beneficiaries, advertisement through social networking platforms and referrals from RASASA’s health and social support workers. Syrian refugees living in Sultanbeyli who gave their consent to participate were screened to assess whether they were eligible to participate in the study. Inclusion criteria were (1) being 18 years or above, (2) being a Syrian with a temporary protection status granted by the government (allowing them to legally stay in Turkey and access basic services), (3) being an Arabic speaker, (4) having elevated levels of psychological stress (score > 15 on the Kessler-10 Psychological Distress Scale) [31, 32] and and (5) having reduced psychosocial functioning determined by scoring higher than 16 on the WHO Disability Scale (WHODAS) [33]. Exclusion criteria were having (1) an acute medical condition, (2) an imminent risk of suicide, (3) severe mental disorder (psychotic disorders or substance use dependence), or (4) severe cognitive impairment (e.g., severe intellectual disability). Inclusion and exclusion criteria were determined by the STRENGHTS consortium [30] to establish a common method for all country partners and were adapted to the Turkey site. The recommended assessment tools as suggested in the PM+ manual was used for the screening assessments. Participants who were excluded from the study at this stage were referred to appropriate treatment or support services.

Eligible participants who have provided informed consent to participate in the study attended baseline assessment right after screening. Screening and baseline assessments were conducted on 22–23 September 2018 by an Arabic-speaking team of trained assessors. Post-assessments were conducted 1-week after and follow-up assessments were conducted 3-month after the fifth and final session of gPM+.

### Procedure

#### Randomisation and blinding

Eligible participants were randomised into intervention or enhanced usual care (E-CAU) with a 1:1 ratio by an independent researcher who was not involved in the study. The intervention arm received gPM+ and E-CAU while the control arm received E-CAU only. Randomisation was conducted by allocating participants into two arms using a computer-generated random-number list. Participants could not be blinded to their study arm because of the nature of the intervention, but outcome assessors were blinded.

#### Intervention

The intervention used in the study was gPM+ and all PM+ materials were linguistically and culturally adapted to make them suitable for Syrian populations [34]. gPM+ was delivered by non-specialist Arabic-speaking facilitators who were peer refugees. Peer refugees were eligible to be facilitators of gPM+ if they had completed at least 12 years of education. Before the gPM+ delivery, peer refugees had to undergo an 8-day training. Facilitators received weekly local group supervision by certified PM+ trainers. The aim of the supervisions was to supervise and monitor the intervention delivery process and protect the well-being of the facilitators. e PM+ groups consisted of eight to ten participants, separated by gender. Facilitators were matched by gender to the group they were leading, and this was for reasons of cultural sensitivity. Five sessions of gPM+ were delivered over five consecutive weeks. Participants who did not attend more than two sessions were counted as drop-outs. Treatment completion was defined as having attended three or more sessions.

#### Enhanced care as usual

Usual care (free access to health services in primary health care centres and hospitals in addition to services provided by migrant health care centres) was enhanced by providing Syrian refugees with a leaflet that included information on available community mental health services that were delivered in Arabic.

#### Process evaluation

To assess acceptability, feasibility, and perceived impact of gPM+ from different perspectives, we conducted semi-structured interviews. Seventeen persons from different stakeholder groups (five gPM+ participants who completed all gPM+ sessions, five participants who dropped-out, five family members of participants who completed gPM+, and two gPM+ facilitators) were interviewed. Purposive sampling was used to achieve a heterogenous sample in terms of key characteristics of the respondents (i.e., gender and age) (see Appendix 4 for further information on respondents). The participants who agreed to participate and gave their consent were invited to RASASA for the interview. One topic guide was developed which focused on the acceptability, feasibility, and perceived impact of gPM+, and this topic guide was tailored to the specific stakeholder group which were being interviewed (gPM+ participants, their family members and the facilitators) (see Appendix 3 for the gPM+ participant’s topic guide). These interviews (45 to 60 min in length) were conducted by a team of research assistants that consisted of one interviewer and two note-takers. The interviews were not audio recorded for confidentiality reasons and the two note-takers were responsible from writing the responses of the participants in verbatim. Interviewers were trained on the qualitative assessment methods and study purposes.

Fidelity to intervention delivery was assessed with a checklist by Arabic speaking field coordinators who were trained on gPM+. According to previous studies and recommendations for PM+, around 10% of the sessions were selected for fidelity assessments [25, 35]. The coordinators were responsible from attending the selected sessions and observing the session while completing the fidelity checklist for that session. The checklist included the criteria for following the necessary steps that are specified in the gPM+ manual for the facilitators.

#### Outcome measures

The outcome measures were agreed upon by the STRENGTHS consortium [30] to establish a common method for all country partners and were further explained in the protocol paper for the current study [36]. The primary outcome measure was the Hopkins Symptoms Checklist (HSCL-25) at 3-month follow up [37]. Secondary outcome measures were the PTSD Checklist for DSM-5 (PCL-5) [38], the Psychological Outcomes Profiles Scale (PSYCHLOPS) [39], and the Client Service Receipt Inventory (CSRI). Other measures were a lifetime trauma exposure questionnaire developed specifically for this project and the Post-Migration Living Difficulties Checklist (PMLDC) [40, 41]. Outcome measures were administered to assess applicability in advance of the fully-powered RCT. They are further detailed in Appendix 2.

#### Economic impacts

Data on health service utilisation and lost employment productivity, for participants themselves or family members providing informal care, were measured using a modified version of the Client Service Receipt Inventory (CSRI) [27] designed for STRENGTHS and adapted to the Turkish context. Our CSRI recorded information on the frequency and duration of service contacts; appropriate unit costs for services in Turkey were then attached to estimate health system costs from the perspective of the publicly funded health insurance system, in addition to productivity costs to society valued using the Minimum Wage Determination Commission set national minimum wage in 2020. All costs are reported in 2020 Turkish Lira in Appendix 7, which provides information on unit costs and their sources.

#### Qualitative and quantitative analyses

We followed certain steps during the qualitative analysis according to framework analyses [42]. First, the verbatim notes of the qualitative interviews that were conducted in Arabic were translated into English by bi-lingual research assistants. Second, we familiarized ourselves with the interviews and recorded reflective notes on its content. Third, we started coding interviews. To ensure that important aspects to the interest of this study were not missed we primarily used deductive coding but did also use some inductive approaches to take account of emerging issues which we did not consider initially (e.g., codes generated from the topic guide were the content of PM+ and management of the group, and codes generated inductively were facilitators and barriers under the sub-theme of implementation of the skills and strategies). After several rounds of coding, researchers who have coded the transcripts met to compare their coding. Their probability of agreement was 90.6% and Cohen’s Kappa was 0.8. Fourth, we finalized an analytical framework with codes being grouped into categories and then data of all interviews was charted to the framework matrix. Fifth, data was interpreted subsequently and differences and characteristics of data were identified.

Quantitative data was analysed using IBM SPSS 21.0 package software program [43]. Analyses were conducted on an intent-to-treat basis. To observe the possible changes/alteration in the condition of the participants, we used linear-mixed models for continuous outcome variables with fixed effects for group, time and group x time interaction term and random effect of subject. The mean differences of treatment and control group at each outcome assessment time point were calculated by considering 95% confidence intervals. No imputation of missing data was conducted because multilevel models can handle missing data [44].

For the economic analysis, mean differences in the economic costs and use of health services, as well as in productivity losses between baseline and 3-month follow up between the two groups were analysed and uncertainty in cost distribution was accounted for using bias-corrected and accelerated bootstrapping.

## Results

**Fig. 1:**
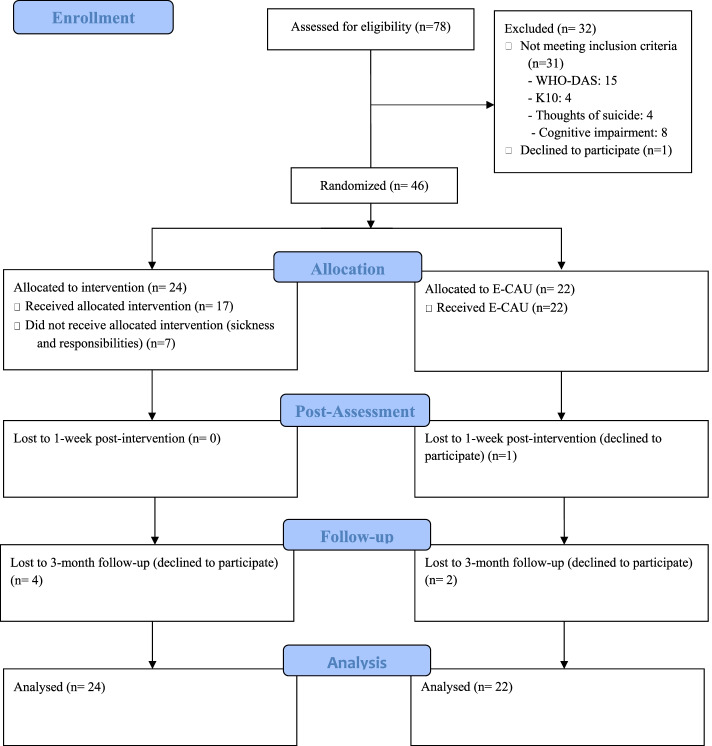
Consort Flow Diagram

In total, 78 potential participants were assessed for eligibility. Of these, 46 participants were included and randomised into gPM + (*n* = 24) or E-CAU (*n* = 22). Figure 1 presents the CONSORT flow diagram.

The characteristics of the sample are given in Table 1. There was no significant group difference on demographic variables, post-migration living difficulties, traumatic experiences, and outcome variables between the intervention and control groups at baseline.

**Table 1:** Sample Characteristics

	Total Sample (*N* = 46)	PM+/E-CAU (*N* = 24)	E-CAU (*N* = 22)
Gender (female) n (%)	31 (67.4%)	16 (66.7%)	15 (68.2%)
Age M (SD)	38.02 (10.88)	37.59 (12.93)	38.42 (8.87)
Marital Status n (%)			
Married	37 (80.4%)	21 (87.5%)	16 (72.7%)
Widowed	4 (8.7%)	1 (4.2%)	3 (13.6%)
Never married	3 (6.5%)	1 (4.2%)	2 (9.1%)
Divorced	1 (2.2%)	1 (4.2%)	0 (0%)
Separated	1 (2.2%)	0 (0%)	1 (4.5%)
Education n (%)			
No or basic education	32 (69.6%)	18 (75%)	14 (63.6%)
Higher education (bachelor’s and master’s degree)	7 (15.2%)	3 (12.5%)	4 (18.2%)
Vocational or other secondary education	7 (15.2%)	3 (12.5%)	4 (18.2%)
Post-migration Living Difficulties M(S.D.)	1.91 (0.75)	1.98 (0.80)	1.84 (0.72)
Traumatic Experiences			
Being a civilian in a war zone	39 (84.78%)	19 (79.2%)	20 (90.9%)
Being in danger during the flight	31 (67.39%)	17 (70.8%)	14 (63.6%)
Serious accident, fire, or explosion	27 (58.7%)	10 (41.7%)	17 (77.3%)
Lack of food or water	27 (58.7%)	13 (54.2%)	14 (63.6%)
Unnatural death of a family member or friend	27 (58.7%)	15 (62.5%)	12 (54.5%)

### Attendance, follow-up and fidelity

Three groups (2 female and 1 male group) were in the intervention arm for the delivery of gPM+. 75% of the 24 participants who were randomised to gPM+ completed the treatment. Common reasons for not attending sessions were sickness, lack of time and no approval from their employer to attend sessions. Data were collected from 40 participants at 1-week post-intervention and 3-month follow-up (Fig. 1). No serious adverse events occurred in our study. 13% of sessions were assessed using the gPM+ fidelity checklist and indicated that 80% of the core components of gPM+ were delivered well and adhered to and that 20% of core components (e.g., managing stress exercise and using group facilitation skills) were only delivered partially. The fidelity checklist can be obtained from the authors upon request.

### Acceptability of gPM+ and feasibility of attendance

Generally, the content of gPM+ was perceived as acceptable. The participants reported that the strategies provided in the program and the format (e.g., the format of the sessions as group sessions, the fact that the providers were peer facilitators, provision of the program in Arabic) were acceptable. The participant’s view on the content of gPM+ was generally positive and they described the programme as good and beneficial experience (Q1.2–5,^1^ Q1.6, Q1. 8). One of the participants stated that “My situation is really bad, this course helped me very much to deal with my life and psychological problems.” (Q1.6). The participants particularly emphasised the benefits of two gPM+ strategies, namely stress management and problem solving (Q1.1 & Q1.7). Participants were able to enact the skills and strategies that they learned in the sessions, for example, by making “*plans*” for each problem and dividing the plans into steps (Q1.9–12, Q1.16, Q1.20–21) and by managing their stress through the strategies they learned from the programme (Q1.13–14, Q1.16–19, Q1.22, Q1.23–28). Only one participant mentioned a barrier to practicing the strategies further after the sessions which was lack of time due to work constraints (Q1.31).

As presented further in Appendix 5, participants stated that the support of their family members made it more feasible for them to attend the sessions (Q1.62–65, Q1.68–69, Q1.72). One participant stated that “The existence of my family and husband facilitate attendance and motivate me.” (Q1.64). However, one of the relatives of a participant mentioned that the participant had difficulty in leaving his children to attend the sessions (Q1.75). The overall impressions of the relatives of the gPM+ participants were positive and they have found the program beneficial for their family member (Q1.29–30).

The main challenges from the participants’ perspective related to attending the sessions were the timing of the sessions in relation to work requirements (Q1.61, Q1.73), the overall length and duration of the programme (Q1.60–61, Q1.66–67), child care responsibilities (Q1.71, Q1.74–75), and a feeling of embarrassment if people were to find out that they were attending a psychosocial programme (Q1.70).

### Group format and facilitators

The perceived benefits of gPM+ were the opportunity to share problems and concerns with each other in a group (Q1.32–37, Q1.40–5). One participant reported that “When listening to other people’s problems, it helps to solve my own (problems).” (Q1.35). Moreover, meeting new people was stated as an advantage of the group format (Q1.37–39, Q1.42). However, two participants also spoke about the challenges of the group format. For example, one participant stated that listening to other problems of participants induced stress (Q1.43) while another participant stopped attending the programme as their problems were perceived as minor compared to others (Q1.42). Participants’ views on the facilitators were generally positive. Participants enjoyed the skills of facilitators in managing the group (Q1.44–46), their overall interaction with the group and approach (Q1.50–51, Q1. 52, Q1. 56, Q1.58), cultural sensitivity, use of informal Arabic language (Q1.47), their way of introducing strategies (Q1.48–49, Q1.52, Q1.54), and helping participants to understand the strategies (Q1.55–56, Q1.58).

### Experience of delivering group gPM+

Facilitators reported that delivery of gPM+ was facilitated by the illustrations (Q2.1), case examples (Q2.2), and exercises to practice skills (Q2.3) which were included in the manual. Barriers from their perspective were participant-related factors such as having prejudices about the programme (Q2.9); low adherence due to some participants not attending sessions (Q2.12–14); and management of the group such as the difficulty to reconcile different opinions from participants (Q2.15). Further information and quotes of the qualitative anlyses are included in Appendix 5.

### Treatment effect

The linear-mixed model analyses of primary and secondary outcomes are described in Table 2. Results showed a significant effect of time for HSCL-25, *F* (2, 82) = 4.75, *p* = .011, *d* = .42. HSCL-25 scores significantly decreased from baseline (*M* = 2.34, *SD* = 0.60) to post-assessment (*M* = 2.15, SD = 0.60), t (82) = 2.43, *p* = .02, d = .36, and to follow-up (*M* = 2.11, *SD* = 0.59), *t* (83) = 2.83, *p* = .02, *d* = .42. There was no significant difference between post (*M* = 2.15, *SD* = 0.60) and follow-up assessment (*M* = 2.11, *SD* = 0.47), *t* (82) = .49, *p* = .63, *d* = .07. Neither the effect of group (*F* (1, 43) = 0.42, *p* = .52, d = .13) nor the interaction between time and group was significant (*F* (2, 82) = 2.28, *p* = .11, *d* = .23).

**Table 2:** Results from mixed-model analysis of primary and secondary outcomes

		Descriptive statistics, M (SD)		Mixed-model analysis		
Outcomes	Time point	gPM+ (*n*=24)	E-CAU (*n* = 22)	Mean differences	*p*-value	Effect size
HSCL-25 total	Baseline	2.37 (0.58)	2.31 (0.64)			
	Post-assessment	2.01 (0.59)	2.28 (0.58)	0.27 (0.26**—**0.29)	0.109	0.48
	Follow-up	2.07 (0.52)	2.14 (0.43)	0.07 (0.06**—**0.08)	0.698	0.12
PCL-5	Baseline	1.84 (0.88)	1.70 (0.86)			
	Post-assessment	1.27 (0.70)	1.59 (0.86)	0.32 (0.31**—**0.34)	0.185	0.40
	Follow-up	1.12 (0.85)	1.26 (0.70)	0.14 (0.13**—**0.16)	0.553	0.18
PSYCHLOPS	Baseline	4.20 (0.68)	3.92 (0.84)			
	Post-assessment	3.82 (1.00)	3.82 (0.63)	0.00 (-0.10**—**0.10)	0.996	0.00
	Follow-up	3.67 (1.32)	3.23 (1.63)	-0.44 (-0.41**—**(-0.48))	0.319	-0.30

*M* Mean, *SD* standard deviation, *HSCL-25* 25-item Hopkins Symptoms Checklist, *PCL-5* PTSD Checklist for DSM-5, *PSYCHLOPS* Psychological Outcomes Profiles

For PCL-5, the effect of time was significant, *F* (2, 88) = 10.01, *p* = .000, *d* = .66. PCL-5 scores significantly decreased from baseline (*M* = 1.77, *SD* = 0.86) to post-assessment (*M* = 1.43, *SD* = 0.79), *t* (88) = 2.61, *p* = .011, *d* = .39, and to follow-up (*M* = 1.19, *SD* = 0.78), *t* (88) = 4.45, *p* = .00, *d* = .66. There was no significant difference in PCL-5 scores between post-assessment (*M* = 1.43, *SD* = 0.79) and follow-up (*M* = 1.19, *SD* = 0.78), *t* (88) = 1.85, *p* = .07, *d* = .27. The effect of group on PCL-5 scores was not significant, *F* (1, 44) = 0.34, *p* = .56, *d* = .31. The interaction effect between time and group was not observed, *F* (2, 88) = 1.56, *p* = .22, *d* = .01.

For PSYCHLOPS, results showed a significant effect of time, *F* (2, 34) = 3.448, *p* = .043, *d* = .34. Self-identified problems significantly decreased from baseline (*M* = 4.06, *SD* = 0.76) to follow-up (*M* = 3.45, *SD* = 1.32), *t* (38) = 2.57, *p* = .04, *d* = .34, but not from baseline to post-assessment (*M* = 3.82, *SD* = 0.86), *t* (37) = 1.01, *p* = .95, *d* = .15, and from post-assessment to follow-up, *t* (37) = 1.33, *p* = .57, *d* = .20. There was no significant effect of group on self-identified problems, *F* (1, 37) = 0.73, *p* = .40, *d* = .25. The effect of interaction between time and group was also not observed, *F* (2, 34) = 0.34, *p* = .72, *d* = .12.

### Economic analyses

Table 3 provides information on mean utilisation of health services, as well as mean lost productivity loss days and their costs. The feasibility study demonstrated that the CSRI was well completed and should provide substantial information to inform cost-effectiveness analysis in a subsequent fully-powered trial. The analysis revealed that there was no significant difference in overall cost between the two groups nor in any single element of costs to the health system or productivity losses. The detailed results of the economic analyses are provided in Appendix 6.

**Table 3:** Service Utilisation

Service (unit of measurement)	Baseline		Post-assessment		3 MFU	
	PM+ (*n*=24)	ETAU (*n*=22)	PM+ (*n*=24)	ETAU *n*=21)	PM+ (*n*=20)	ETAU (*n*=20)
Community health worker (contact)	0.04 (0.20)	0.36 (1.34)	0.00	0.05 (0.21)	0.08 (0.41)	0.36 (0.79)
Community-based doctor (contact)	0.96 (2.54)	0.59 (1.05)	0.25 (1.03)	0.23 (1.06)	0.50 (1.29)	0.86 (1.58)
Psychiatrist (contact)	0.00	0.91 (4.26)	0.00	0.05 (0.21)	0.42 (2.04)	0.00
Psychologist (contact)	0.00	0.00	0.00	0.00	0.04 (0.20)	0.00
Psychiatric Nurse (contact)	0.00	0.00	0.00	0.00	0.00	0.00
Social worker (contact)	0.00	0.05 (0.21)	0.00	0.00	0.00	0.09 (0.43)
Psychiatric inpatient stay (nights)	0.00	0.00	0.00	0.00	0.00	0.09 (0.43)
Other inpatient stay (nights)	0.25 (1.22)	0.05 (0.21)	0.92 (4.07)	1.32 (4.02)	0.08 (0.28)	0.23 (0.53)
Hospital Emergency Department (contact)	0.25 (0.74)	0.55 (2.13)	0.29 (0.91)	0.00	0.00	0.77 (2.78)
Psychiatric outpatient (contact)	0.00	0.00	0.00	0.05 (0.21)	0.00	0.00
Other outpatient (contact)	1.29 (2.74)*	0.09 (0.43)*	0.00	0.00	0.54 (1.91)	0.14 (0.47)
Day Hospital (Visit)	0.00	0.68 (1.86)	0.42 (2.04)	0.00	0.00	0.59 (2.56)
Policlinic (Visit)	0.29 (1.08)	0.36 (1.50)	0.29 (1.08)	0.36 (1.50)	0.17 (0.64)	0.45 (1.47)
Medicine (doses)	7.58 (37.15)	0.00	3.79 (18.57)	0.00	3.96 (18.97)	13.00 (59.57)
CAM (contact)	0.42 (1.47)	0.18 (0.59)	0.00	0.00	0.00	0.14 (0.64)
Productivity Loss (days)	8.71 (19.00)	2.55 (6.72)	6.04 (8.89)	12.36 (50.89)	0.54 (1.44)	3.50 (10.37)

Mean utilisation (SD) at baseline, post-assessment and three month follow up. (complete cases only – no imputed data)

* *p* < 0.05

Able to collect CSRI data on contacts with services; low use of services for most individuals; no significant differences between utilisation rates at each time point for all categories (using parametric test) other than for other outpatient contacts at baseline

## Discussion

This study was an individual randomised single-blind pilot RCT that aimed to assess feasibility, acceptability, perceived impact and cost-consequences of gPM+ among adult Syrian refugees residing in Turkey. The study was not powered to show an effect and was primarily conducted to assess process indicators (attendance, follow-up, fidelity), acceptability and feasibility of delivery from multiple perspectives. The results of the study indicate that the culturally adapted version of gPM+ provided by non-specialist peer providers is acceptable to participants. The process evaluation showed that Syrian refugees generally had a positive view of the content, implementation and the format of gPM+. Qualitative analyses suggest that gPM+ is feasible to implement from the perspective of the facilitator, and intervention fidelity and attendance rates support this finding. gPM+ seems to be a safe intervention and no adverse events did occur during the pilot. These results are congruent with findings recently reported in the Netherlands in which acceptability and feasibility of the individual version of PM+ was investigated with Syrian refugees [35, 39].

We did not detect any barriers during the recruitment of participants. Partnering with an NGO that work with Syrian refugees greatly facilitated the recruitment process. Retention rates of post and follow-up assessments were also comparable to other studies which have used PM+ previously [24].

Our sample size was small and we did not find significant differences in the primary and secondary outcomes between gPM+ and the E-CAU group at 3-month follow up. However, though not significant, results indicated an improvement in depression, anxiety, PTSD, and self-identified problems scores from baseline to post-treatment and/or follow up in both groups. Additionally, it is important to note that there was a trend for a significant decrease in PTSD scores from post-treatment to follow-up which pointing out that these scores might continue to decrease. In a larger study, this trend might appear to be significant. Furthermore, except for self-identified problems, those in the gPM+ group seemed to have fewer symptoms than those in the E-CAU group at the post treatment and 3-month follow up assessments, which is similar to previous studies on PM+ among conflict-affected populations [25, 45, 46]. The economic analysis focused on the cost consequences of participation in gPM+ group compared to E-CAU. We demonstrated that the adapted CSRI for Syrian refugees in Turkey could be used to collect this data, and that our study population appeared to have little prior or ongoing contact with specialist mental health services. No significant differences in health system costs or in productivity losses between the two groups were found; and a longer term follow up in a fully-powered trial is needed to assess improvements in mental health status and potential cost-effectiveness of gPM+. No significant differences in health system costs or in productivity losses between the two groups were found; and a longer term follow up in a fully powered trial is needed to assess improvements in mental health status and determine whether mental health gains achieved, with costs of delivering gPM+ of approximately 380 TL per participant, are potentially cost effective. It is also important to recognise the complexity of the health system in Turkey, even after major reforms which have consolidated several insurance funds into one public insurance system and ensured free of point access to primary care [47]; further data collected using the CSRI also indicates that some refugees still incur additional out of pocket costs for privately funded services. For example, 5 study participants (11%) had out of pocket costs of up to 220 Turkish Lira per community doctor consultation. Future analysis at scale could also explore potential differences in outcomes taking account of these differences.

This study was the first pilot RCT that assessed the feasibility of gPM+ with Syrian refugees in Turkey. A major strength of this study was that we could demonstrate that the methodological procedures of our study can be built on in a fully-powered RCT, and that participants and facilitators found our intervention acceptable and feasible. We conducted a range of interviews with different stakeholders, including gPM+ completers and non-completers. This allowed us to explore the content of gPM+, implementation of strategies and skills, and feasibility of attending the sessions from the perspectives of those who completed the programme and who discontinued for various reasons. Moreover, we included family members of the participants to investigate the perceived impact of the programme on the interpersonal interactions within families.

There are a few interventions conducted in Turkey that are delivered in a peer-to-peer format, however, most of them are educational interventions and do not focus on mental health [48, 49]. To the best of our knowledge, this is the first study using a psychosocial intervention delivered by peer-refugees in Turkey and we have shown that peer refugees who do not have a background in providing mental health interventions can be effectively trained to deliver gPM+.

There were a few limitations of our study. The qualitative interviews were not audio recorded as this is not allowed in Turkey and had to be transcribed by note-takers during the interviews. Although recorded transcripts and interview notes are shown to be comparable [50], some information provided during the interviews might have been missed. In addition, participants from both arms generally provided positive feedback on the intervention and this might be due to the fact there were three research assistants present during the interview for note taking. It is possible that some information such as negative experiences related to the intervention and critiques were not raised by participants and that there was a tendency towards social expectancy which may have biased the qualitative findings. The Arabic speaking field coordinators who have completed the fidelity checklists have reported that facilitators only partially delivered the managing stress exercise and group facilitation skills strategies. Those two components need to be strengthened in the training of facilitators in the future. Finally, our study was not powered to show an effect on primary and secondary outcomes measures as this was not the purpose of our study.

## Conclusion

The results of this study indicate that the methodological procedures, including recruitment, retention and attendance to gPM+ sessions are promising and can be built on in a fully-powered RCT. Overall, gPM+ seems to be acceptable for participants and feasible for facilitators to deliver. Different formats of PM+ are currently being evaluated with Syrian refugees in various other countries within the STRENTHS project [30]. This pool of evidence will add to the body of research on PM+, and will further support the implementation and dissemination of this brief psychological intervention among Syrian refugees.

## Supplementary Information

**Additional file 1.**

## Data Availability

The datasets analysed during this study are available from the corresponding author, Koc University, Istanbul, Turkey, on reasonable request.

## Footnotes

1 The number right next to “Q” denotes the number of questions while the number after point refers to the number of example quotes. For instance, Q1.1 refers to the first question and first example quote.
