# Supplementary material for: Group problem management plus (PM+) to decrease psychological distress among Syrian refugees in Turkey: a pilot randomised controlled trial
Source: BMC Psychiatry. 2022 Jan 4;22:8. doi: 10.1186/s12888-021-03645-w (PMC8728921; doi:10.1186/s12888-021-03645-w)
Supplement: Supplementary file 1 — Additional file 1. [file 12888_2021_3645_MOESM1_ESM.docx]

# **Appendix**

**Appendix 1:** CONSORT Checklist

| Section/Topic | Item No | Checklist item | Reported on page No |
| --- | --- | --- | --- |
| Title and abstract | | | |
|  | 1a | Identification as a randomised trial in the title | 1 |
|  | 1b | Structured summary of trial design, methods, results, and conclusions (for specific guidance see CONSORT for abstracts) | 3-4 |
| Introduction | | | |
| Background and objectives | 2a | Scientific background and explanation of rationale | 5-7 |
|  | 2b | Specific objectives or hypotheses | 6-7 |
| Methods | | | |
| Trial design | 3a | Description of trial design (such as parallel, factorial) including allocation ratio | 7-8 |
|  | 3b | Important changes to methods after trial commencement (such as eligibility criteria), with reasons | N/A |
| Participants | 4a | Eligibility criteria for participants | 7-8 |
|  | 4b | Settings and locations where the data were collected | 7 |
| Interventions | 5 | The interventions for each group with sufficient details to allow replication, including how and when they were actually administered | 8-9 |
| Outcomes | 6a | Completely defined pre-specified primary and secondary outcome measures, including how and when they were assessed | 10-11, Appendix 2 |
|  | 6b | Any changes to trial outcomes after the trial commenced, with reasons | N/A |
| Sample size | 7a | How sample size was determined | 12 |
|  | 7b | When applicable, explanation of any interim analyses and stopping guidelines | N/A |
| Randomisation: |  |  |  |
| Sequence generation | 8a | Method used to generate the random allocation sequence | 8 |
|  | 8b | Type of randomisation; details of any restriction (such as blocking and block size) | 8 |
| Allocation concealment mechanism | 9 | Mechanism used to implement the random allocation sequence (such as sequentially numbered containers), describing any steps taken to conceal the sequence until interventions were assigned | 8  8 |
| Implementation | 10 | Who generated the random allocation sequence, who enrolled participants, and who assigned participants to interventions | 8 |
| Blinding | 11a | If done, who was blinded after assignment to interventions (for example, participants, care providers, those assessing outcomes) and how | 8 |
|  | 11b | If relevant, description of the similarity of interventions | N/A |
| Statistical methods | 12a | Statistical methods used to compare groups for primary and secondary outcomes | 11-12 |
|  | 12b | Methods for additional analyses, such as subgroup analyses and adjusted analyses | N/A |
| Results | | | |
| Participant flow (a diagram is strongly recommended) | 13a | For each group, the numbers of participants who were randomly assigned, received intended treatment, and were analysed for the primary outcome | Figure 1 |
|  | 13b | For each group, losses and exclusions after randomisation, together with reasons | Figure 1 |
| Recruitment | 14a | Dates defining the periods of recruitment and follow-up | 8 |
|  | 14b | Why the trial ended or was stopped | N/A |
| Baseline data | 15 | A table showing baseline demographic and clinical characteristics for each group | Table 1 |
| Numbers analysed | 16 | For each group, number of participants (denominator) included in each analysis and whether the analysis was by original assigned groups | 12, Figure 1 |
| Outcomes and estimation | 17a | For each primary and secondary outcome, results for each group, and the estimated effect size and its precision (such as 95% confidence interval) | 12, Appendix 5 |
|  | 17b | For binary outcomes, presentation of both absolute and relative effect sizes is recommended | N/A |
| Ancillary analyses | 18 | Results of any other analyses performed, including subgroup analyses and adjusted analyses, distinguishing pre-specified from exploratory | 16, Appendix 6 |
| Harms | 19 | All important harms or unintended effects in each group (for specific guidance see CONSORT for harms) | N/A |
| Discussion | | | |
| Limitations | 20 | Trial limitations, addressing sources of potential bias, imprecision, and, if relevant, multiplicity of analyses | 19 |
| Generalisability | 21 | Generalisability (external validity, applicability) of the trial findings | 19-20 |
| Interpretation | 22 | Interpretation consistent with results, balancing benefits and harms, and considering other relevant evidence | 16-19 |
| Other information | | |  |
| Registration | 23 | Registration number and name of trial registry | 4 |
| Protocol | 24 | Where the full trial protocol can be accessed, if available | 4 |
| Funding | 25 | Sources of funding and other support (such as supply of drugs), role of funders | 20 |

**Appendix 2:** The Study Outcome Measures

Demographic information was collected and psychological functioning was measured by WHO Disability Assessment Schedule 2.0 (WHODAS 2.0)[1]. The demographic section of the questionnaire consisted of 10 and the psychological functioning section of the questionnaire consisted of 15 items asking about difficulties people experience in different domains of life (cognition, mobility, self-care, getting along, life activities, and participation) considering the last 30 days. First 12 items were rated between 1 (none) and 5 (cannot do) which total score ranged from 12 to 60. Last 3 questions were about the number of days participants experienced certain difficulties. Higher scores indicated difficulties in functioning. The WHODAS 2.0 was validated in different cultures [2]. For the purpose of this study, this measure was used as a screening tool and the threshold score for inclusion was determined as 16 points.

Psychological distress was measured by the Kessler-10 Psychological Distress Scale [3]. This questionnaire includes 10 items asking about various aspects of distress such as being nervous, feeling hopeless and worthless. Each item was rated between 1 (none of the time) and 5 (all of the time) which total score ranged between 10 and 50. Higher scores indicated more psychological distress. The Arabic version of K-10 was validated for Afghan and Kurdish refugee groups [4]. For the purpose of this study, this measure was used as a screening tool and the threshold score for inclusion was determined as 15 points.

The other two screening tools that were used for this study were the questionnaire on thoughts of suicide and the questionnaire on cognitive, mental, neurological impairments or substance use disorders. These tools were from the Group PM+ intervention manual [5].

The primary outcome measure was The Hopkins Symptoms Checklist (HSCL-25) [6, 7]. HSCL-25 consists of 25 items and divided to three subscales which are on depression symptoms (13 items), anxiety symptoms (10 items) and somatic symptoms (2 items). Each item was rated between 1 (not at all) and 4 (extremely). Mean scores will be used. The validated cut-off score of 1.75 [6, 8] will be used for anxiety and somatic subscales and the validated cut-off score of 2.1 will be used for the depression subscale [9]. The Arabic version of HSCL-25 was used in various studies [10–13].

The secondary outcome measures consisted of four measures. Post-traumatic stress disorder symptoms were assessed with the PTSD Checklist for DSM-5 (PCL-5) [14]. PCL-5 consists of 20 items asking about the symptoms of traumatic stress such as intrusion and avoidance symptoms, negative mood and cognitions and alterations in arousal and reactivity. Each item is rated between 0 (not at all) and 4 (extremely) which total score ranges between 0 and 80. Mean scores will be used. Higher scores indicated increased symptoms of post-traumatic stress. The Arabic version of PCL-5 was used in various studies [15, 16].

Self-identified problems were assessed with The Psychological Outcomes Profiles (PSYCHLOPS) scale [17]. PSYCHOLOPS consists of four questions, three domains (problems, functions and well-being) and it assesses the change after the intervention. Participants were asked to answer open-ended questions about their self-identified problems and function domains. The answers were scored between 0 (not at all) and 5 (severely). Higher scores mean higher self-identified problems. Means scores will be used. The PSYCHOLOPS was validated in various cultures [18, 19].

Mental health service utilization and the cost of care were measured with CSRI schedule to calculate the economic impacts on service utilisation and productivity loss of intervention provision. This scale was developed for the study and translated according to the WHO guidelines by VU Amsterdam research team.

Access to health care was measured with a questionnaire that consisted of 23 items about the use of mental health services and the reasons for not using these services. This scale was developed for the study and translated according to the WHO guidelines by VU Amsterdam research team.

There were other measures used in the study. The lifetime trauma exposure was measured with a questionnaire that was developed for the study. This questionnaire included items from the Harvard Trauma Questionnaire (HTQ) and the Post-traumatic Diagnostic Scale (PDS). It had 28 items that asked about various traumatic events such as serious injury, being in a warzone, kidnapped and tortured. Each item was rated as either 0 (no) or 1 (yes) which the total score ranged between 0 and 28. Higher scores indicated higher number of different traumatic events experienced by the participant.

Post-migration stressors were assessed with The Post-Migration Living Difficulties Checklist (PMLD) [20, 21]. PMLD consists of 17 items about various stressors such as discrimination, communication difficulties, difficulties obtaining financial assistance. Each item was rated between 0 (not a problem) and 4 (very serious problem) which the total score ranged between 0 and 68. Higher scores increased post-migration problems experienced by the participants. The Arabic version of the scale was used in studies before [22].

**References:**

1. Üstün TB, Chatterji S, Kostanjsek N, Rehm J, Kennedy C, Epping-Jordan J, et al. Developing the world health organization disability assessment schedule 2.0. Bull World Health Organ. 2010;88:815–23.

2. Üstün TB. Measuring Health and Disability: Manual for WHO Disability Assessment Schedule WHODAS 2.0. 2010.

3. Kessler RC, Andrews G, Colpe LJ, Hiripi E, Mroczek DK, Normand SLT, et al. Short screening scales to monitor population prevalences and trends in non-specific psychological distress. Psychol Med. 2002;32:959–76.

4. Sulaiman-Hill CM, Thompson SC. Selecting instruments for assessing psychological wellbeing in Afghan and Kurdish refugee groups. BMC Res Notes. 2010;3:1–9.

5. Group Problem Management Plus (Group PM+). World Health Organization. 2020. https://www.who.int/publications/i/item/9789240008106.

6. Mollica RF, Wyshak G, de Marneffe D, Khuon F, Lavelle J. Indochinese versions of the Hopkins Symptom Checklist-25: a screening instrument for the psychiatric care of refugees. Am J Psychiatry. 1987;144:497–500.

7. Parloff MB, Kelman HC, Frank JD. Comfort, effectiveness, and self-awareness as criteria of improvement in psychotherapy. Am J Psychiatry. 1954;111:343–52.

8. Nettelbladt P, Hansson L, Stefansson CG, Borgquist L, Nordström G. Test characteristics of the Hopkins Symptom Check List-25 (HSCL-25) in Sweden, using the Present State Examination (PSE-9) as a caseness criterion. Soc Psychiatry Psychiatr Epidemiol. 1993;28:130–3.

9. Mahfoud Z, Kobeissi L, Peters TJ, Araya R, Ghantous Z, Khoury B. The Arabic Validation of the Hopkins Symptoms Checklist-25 against MINI in a Disadvantaged Suburb of Beirut, Lebanon. Int J Educ Psychol Assess. 2013;13:17–33.

10. Al-Turkait FA, Ohaeri JU, El-Abbasi AHM, Naguy A. Relationship between symptoms of anxiety and depression in a sample of Arab college students using the Hopkins Symptom Checklist 25. Psychopathology. 2011;44:230–41.

11. Caspi Y, Saroff O, Suleimani N, Klein E. Trauma exposure and posttraumatic reactions in a community sample of Bedouin members of the Israel Defense Forces. Depress Anxiety. 2008;25:700–7.

12. Selmo P, Koch T, Brand J, Wagner B, Knaevelsrud C. Psychometric properties of the online Arabic versions of BDI-II, HSCL-25, and PDS. Eur J Psychol Assess. 2016;35:46–54.

13. Fares S, Dirani J, Darwish H. Arabic validation of the hopkins symptom checklist-25 (HSCL) in a Lebanese sample of adults and older adults. Curr Psychol. 2019;:1–8.

14. Weathers FW, Litz BT, Keane TM, Palmieri PA, Marx BP, Schnurr PP. The PTSD Checklist for DSM-5 (PCL-5). Natl Cent PTSD. 2013.

15. Thabet AA, Tawahina AA, El Sarraj E, Vostanis P. Exposure to war trauma and PTSD among parents and children in the Gaza strip. Child Adolesc Psychiatry. 2008;17:191–9.

16. Ibrahim H, Ertl V, Catani C, Ismail AA, Neuner F. The validity of Posttraumatic Stress Disorder Checklist for DSM-5 (PCL-5) as screening instrument with Kurdish and Arab displaced populations living in the Kurdistan region of Iraq. BMC Psychiatry. 2018;18:1–8.

17. Ashworth M, Shepherd M, Christey J, Matthews V, Wright K, Parmentier H, et al. A client-generated psychometric instrument: The development of ‘PSYCHLOPS.’ Couns Psychother Res. 2004;4:27–31.

18. Czachowski S, Seed P, Schofield P, Ashworth M. Measuring psychological change during cognitive behaviour therapy in primary care: a Polish study using ‘PSYCHLOPS’(Psychological Outcome Profiles). PLoS One. 2011;6:e27378.

19. Héðinsson H, Kristjánsdóttir H, Ólason DÞ, Sigurðsson JF. A validation and replication study of the patient-generated measure PSYCHLOPS on an Icelandic clinical population. Eur J Psychol Assess. 2013;29:89–95.

20. Silove D, Sinnerbrink I, Field A, Manicavasagar V, Steel Z. Anxiety, depression and PTSD in asylum-seekers: Associations with pre-migration trauma and post-migration stressors. Br J Psychiatry. 1997;170:351–7.

21. Steel Z, Silove D, Bird K, McGorry P, Mohan P. Pathways from war trauma to posttraumatic stress symptoms among Tamil asylum seekers, refugees, and immigrants. J Trauma Stress. 1999;12:421–35.

22. Schick M, Zumwald A, Knöpfli B, Nickerson A, Bryant RA, Schnyder U, Müller J, Morina N. Challenging future, challenging past: The relationship of social integration and psychological impairment in traumatized refugees. European journal of psychotraumatology. 2016;7:28057.

**Appendix 3: Topic Guide for gPM+ Participants**

1. **Greet person.**

**Introduce self, including what organization you are working for.**

1. **Explain the study following written informed consent process.**

**Possible additional explanation of in-depth interview process:**

**“You have recently participated in the Group PM+ program. We would like to ask you some questions about your experience of this program to help us to think about how it could be improved for delivery in the future. There are no right or wrong answers to the questions we are going to ask. We will be speaking to a number of people, asking everyone the same questions. If you feel unable to answer a question please say and we will move on to the next one.”**

1. **If person agrees to be interviewed, find a private location (if not already in one). If person declines to participate thank him/her and leave.**
2. **In exercise book document date and site of interview, age and gender of interviewee, who they are (i.e., Group PM+ participant, family member/friend, Group PM+ providers, policy maker), and initials of interviewers.**
3. **Begin in-depth interview:**

**A) Overall impressions:**

**- Can you describe your experience of the Group PM+ intervention? - Explore positive / negative views through probes.**

**B) Rapport with Group PM+ providers:**

**- Can you describe how you found working with your PM+ provider?**

**- Explore positive / negative views through probes.**

**- How did your family view your relationship with your Group PM+ providers? – Explore positive / negative views through probes.**

**C) Intervention adherence:**

**- Can you describe how easy or difficult you found attending weekly group sessions for five weeks? - Explore barriers and facilitators to attendance.**

**- Can you describe how you found implementing the skills the Group PM+ providers taught to you in your everyday routine?**

**- Explore barriers and facilitators to skills development.**

1. **Review the written record with the interviewee still present. If anything is not clear ask for clarification and correct written notes as necessary.**
2. **Ask the interviewee if they have anything to add. Any additional information is added to the interview notes as required.**
3. **Thank person and leave.**

**Appendix 4: Demographic Information on participants invited for interview.**

| **No** | **Status** | **The reason for not attending** | **Gender** | **Age** |
| --- | --- | --- | --- | --- |
| **P1** | Group PM+ Completer (4 sessions) |  | Male | 58 |
| **P2** | Group PM+ Completer (5 sessions) |  | Male | 34 |
| **P3** | Group PM+ Completer (5 sessions) |  | Female | 24 |
| **P4** | Group PM+ Completer (4 sessions) |  | Female | 33 |
| **P5** | Group PM+ Completer (5 sessions) |  | Female | 36 |
| **D1** | Group PM+ Drop-out (1 session) | Sickness | Male | 46 |
| **D2** | Group PM+ Drop-out (1 session) | Sickness | Male | 44 |
| **D3** | Group PM+ Drop-out (1 session) | Taking care of children | Female | 35 |
| **D4** | Group PM+ Drop-out (1 session) | Family member was sick | Female | 37 |
| **D5** | Group PM+ Drop-out (1 session) | Sickness | Female | 44 |
| **R1** | Group PM+ Participant Relative |  | Female | 46 |
| **R2** | Group PM+ Participant Relative |  | Female | 28 |
| **R3** | Group PM+ Participant Relative |  | Male | 43 |
| **R4** | Group PM+ Participant Relative |  | Male | 30 |
| **R5** | Group PM+ Participant Relative |  | Female | 32 |
| **F1** | Facilitator |  | Male | 23 |
| **F2** | Facilitator |  | Female | 22 |

**Appendix 5:** Categories and related quotes of qualitative data analyses

|  |  | **Interview** | **#** | **Selected Quotes** |
| --- | --- | --- | --- | --- |
| **1: Views on PM+ (from participants and family)** | | | | |
| **1.1 Acceptability of PM+ (from participants and family)** | | | | |
|  | 1.1.1 The content of PM+ | | | |
|  |  | P2 | Q1.1 | I had a general idea of managing my problems before the sessions. However, in the sessions I have learned how to divide the problems to smaller parts then solving each one of them. |
|  |  | P4 | Q1.2 | It was a good experience. |
|  |  | P4 | Q1.3 | The program was important for me. |
|  |  | P4 | Q1.4 | The program in my point of view is very beneficial. |
|  |  | P5 | Q1.5 | The program was beautiful. |
|  |  | D1 | Q1.6 | My situation is really bad, this course helped me very much to deal with my life and psychological problems. |
|  |  | D1 | Q1.7 | The good way of breathing. |
|  |  | D5 | Q1.8 | It was a good and beneficial experience. They provide beneficial information. |
| **1.2 Implementation of the skills and strategies (barriers and facilitators)** | | | | |
|  | 1.2.1 Facilitators | | | |
|  |  | P1 | Q1.9 | Making plans for each problem. |
|  |  | P2 | Q1.10 | After the sessions I became able to solve and manage my problems faster and more easily. |
|  |  | P2 | Q1.11 | After this program, I am now able to listen more to my family's problems and then managing these problems and solving them. |
|  |  | P2 | Q1.12 | I know now how to make a better plan for my own job. |
|  |  | P3 | Q1.13 | Helps passing some of the stress, helped in self-control. |
|  |  | P3 | Q1.14 | I was able to control my nervousness and stress. I was less nervous then before. |
|  |  | P3 | Q1.15 | My husband found an effect on me, and this makes me happy and my psychology improved. |
|  |  | P3 | Q1.16 | I was able to manage my psychological situations and the stress. The techniques like the stress helped a lot and I learned how to define my problem and I am convinced now that each problem can be solved. |
|  |  | P3 | Q1.17 | No extra stess. I learned how to get out from the inactivity cycle. The effect of the program will continue after the program, not only at the period of the program. |
|  |  | P4 | Q1.18 | The change in my angeriness was realized by my family side especially my sister. My sister saw a change which is that my angeriness calmed down. |
|  |  | P4 | Q1.19 | The breath exercise is very helpful since I am a nervous person and I used after the sessions significantly. |
|  |  | P4 | Q1.20 | It (the program) solved some problems significantly, some of my friends were having big problems and they were solved and this is the most important thing and the secret of the success of this program in my opinion. |
|  |  | P5 | Q1.21 | It was a beneficial experience and helped me solving problems. |
|  |  | P5 | Q1.22 | Controlling the stress (a positive point). |
|  |  | P5 | Q1.23 | I became comfortable with the stress exercise and I am still doing it but the other exercise for solving the problem, I am not. |
|  |  | D1 | Q1.24 | It makes us a little bit calm. |
|  |  | D1 | Q1.25 | I became able to limit and decrease my concerns and I am having a good psychological health. |
|  |  | D1 | Q1.26 | It is good for psychological health (a positive point). |
|  |  | D1 | Q1.27 | Breathing exercise made me comfortable. Breathing exercise is good after a long hard day. I was doing it from time to time, my job caused me backache. |
|  |  | D1 | Q1.28 | Breathing exercises are the best things that I have learned from the sessions, it makes me more comfortable. |
|  |  | R1 | Q1.29 | I realized a difference in his psychology, he was coming back from the sessions comfortable. It changed his personality in a good way. |
|  |  | R2 | Q1.30 | He wanted to improve that's why he kept attending the program. The treatment changed. He was nervous, and became calm. His moral changed. He became better and thought his children how to breath. Applying the skills was easy according to him. |
|  | 1.2.2 Barriers | | | |
|  |  | D1 | Q1.31 | My boss in the work is asking me to hurry up with my work. That is why I cannot do some exercises in my work. |
| **1.3 Views on the group format (Group dynamics; Benefits and challenges).** | | | | |
|  | 1.3.1 Benefits | | | |
|  |  | P1 | Q1.32 | Listening to everyone and sharing my personal life and having fun (as a positive point) |
|  |  | P1 | Q1.33 | In the sessions I helped the other participants to understand the idea of the sessions. |
|  |  | P4 | Q1.34 | I was listening to other people's problems so this helped me a lot. |
|  |  | P4 | Q1.35 | The existence of other participants and listening to their problems makes life easier. When listening to other people's problems, it facilitates a lot in solving problems. |
|  |  | P5 | Q1.36 | The discussion in the group helped a lot and we learned from each other, we forget our own problems when we hear the others' ones, taking benefits from the other people's problems. |
|  |  | D1 | Q1.37 | It is good to share our concerns, meeting new people. |
|  |  | D1 | Q1.38 | Meeting new people (a positive point). |
|  |  | D1 | Q1.39 | Communicating with others. |
|  |  | D3 | Q1.40 | When people share their problems, it helps human feeling that his conditions are good, better than the others. |
|  |  | D5 | Q1.41 | Listening to people's problems (a positive point). |
|  |  | R3 | Q1.42 | I made new friends. |
|  | 1.3.2 Challenges | | | |
|  |  | P3 | Q1.43 | One participant was having a problem with her husband and she was sharing her problem a lot which affected me and made me stressed because she was not listening to the provided solutions. |
|  |  | D3 | Q1.44 | I am better than my friends (other participants), I have no problems like them, that is why I stopped attending the sessions. |
| 1.4**Views on the helpers** | | | | |
|  | 1.4.1 Management of the group | | | |
|  |  | P1 | Q1.45 | Interaction with the participants were kind and positive. |
|  |  | D1 | Q1.46 | I liked the way of dealing with us. |
|  | 1.4.2 Culture and language | | | |
|  |  | P1 | Q1.46 | They were using informal Arabic language -the Syrian slang-. The other participants were able to understand it better. |
|  | 1.4.3 Competence | | | |
|  |  | P1 | Q1.48 | The way of teaching was very good. |
|  |  | P2 | Q1.49 | Their way of teaching the new skills was good. |
|  |  | P4 | Q1.50 | The treatment was beautiful. |
|  |  | P4 | Q1.51 | Their treatment was good. |
|  |  | P5 | Q1.52 | Helping in understanding things and repeating if I did not understand (a positive point). |
|  |  | D1 | Q1.53 | Very good, kind and excellent. |
|  |  | D1 | Q1.54 | Their teaching skills are good. |
|  |  | D4 | Q1.55 | The facilitators helped me with the exercises. |
|  |  | D4 | Q1.56 | (The facilitators helped) understanding the sessions. |
|  |  | D5 | Q1.57 | We feel safe and that there is someone caring about us. |
|  |  | D5 | Q1.58 | The facilitators are good. The girls helped me in learning techniques which helped me in my life. |
|  |  | R3 | Q1.59 | They were kind. |
| **1.5 Feasibility of PM+ (view from participants and family)** | | | | |
|  | 1.5.1 Feasibility of attending the sessions | | | |
|  |  | P2 | Q1.60 | It took a long time as weeks for the whole sessions and as hours for each session. |
|  |  | P2 | Q1.61 | It would be better if it was at morning and with less hours. Because we want to have rest in our holidays and go out with our families outside. |
|  |  | P3 | Q1.62 | Family acceptance (as a positive point) |
|  |  | P3 | Q1.63 | No difficulties since it is the weekends, got a help from my family. |
|  |  | P3 | Q1.64 | The existence of my family and husband facilitate and motivate me. |
|  |  | P4 | Q1.66 | There was no problem from the family side in attending the program. My husband knew about the program, no problems. |
|  |  | P4 | Q1.66 | The period was long, if the session was for a short time, it would be better. |
|  |  | P5 | Q1.67 | (When I got back home) I feel like I am tired since its period was long and because I have things to do with my children. |
|  |  | P5 | Q1.68 | My family and husband accepted in attending the sessions. |
|  |  | P5 | Q1.69 | My husband encouraged me to attend. |
|  |  | P5 | Q1.70 | Being embarrassed when telling people about the program and that when they do not accept (negative point). |
|  |  | P5 | Q1.71 | Difficulty of attending due to the presence of children (a barrier). |
|  |  | P5 | Q1.72 | My husband's support and his encouragement (facilitator). |
|  |  | P5 | Q1.73 | The time is long toward our other works on Sunday. |
|  |  | D3 | Q1.74 | No problems with attending, but I could not find enough time since Sunday is the only day for dedicated to my children. |
|  |  | R2 | Q1.75 | He was having some difficulties since he was leaving her and his children and family alone at home to attend the sessions. |
| **2: Experiences of delivering Group PM+ (view from helper)** | | | | |
| **2.1 Facilitators of PM+ delivery** | | | | |
|  | 2.1.1 The Content of PM+ | | | |
|  |  | F1 | Q2.1 | Even they were not able to read or write, but the illustrations helped a bit although they were not good. |
|  |  | F1 | Q2.2 | The case examples help |
|  |  | F2 | Q2.3 | Participants stay active and be motivated when they do the exercises in their house and feel they changed something and found a benefit. |
|  | 2.1.2 The Helper of PM+ | | | |
|  |  | F1 | Q2.4 | Our relationship was between friends and professional, and this helped a lot and we did not find any difficulty in that. At the beginning they were very formal, but then we knew each other more so they were opened up. |
|  |  | F1 | Q2.5 | I think the most things that they benefited from was that they have the chance to talk, and someone listening to them. |
|  |  | F2 | Q2.6 | If the facilitator is more engaged as well that would make participants to come. |
|  | 2.1.3 The Group Format | | | |
|  |  | F2 | Q2.7 | My group was very engaged during the session |
|  |  | F2 | Q2.8 | Also, if there was a friendship between them, a link they have between each other, that would make them motivate each other. |
| **2.2 Barriers to PM+ delivery** | | | | |
|  | 2.2.1 Participant related factors | | | |
|  |  | F1 | Q2.9 | There was some stress since they were thinking that we are coming (to the NGO that the sessions were hold) to manipulate them, and some of them apologized for not coming. |
|  | 2.2.2 The Content of PM+ | | | |
|  |  | F1 | Q2.10 | In general, the sessions are not in the recommended level that people need. |
|  |  | F1 | Q2.11 | The program is a little weak, not very suitable for participants since the peoples’ level of education is low. |
|  | 2.2.3 Adherence | | | |
|  |  | F1 | Q2.12 | Even some of them were refusing coming to the assessment or sessions and they do not want anyone to ask them such questions. |
|  |  | F2 | Q2.13 | There was an absence rate. |
|  |  | F2 | Q2.14 | The thing that prevents them of coming to the session is their husbands. |
|  | 2.2.4 Management of the group | | | |
|  |  | F1 | Q2.15 | We had some problems when there are differences in opinions. |

P: Participant, D: Drop-out, R: Relative, F: Facilitator

**Appendix 6:**  Economic analyses

There were no significant differences at baseline between the two groups, with the exception of non-mental health related outpatient contacts which were significantly greater in the treatment group (p<0.05). Participants reported almost no contact with any specialist mental health services in either group, with the most frequently used services being for contacts with community doctors and general outpatient services. Appendices 8 and 9 compare changes in service utilisation, productivity losses and their costs between baseline and 3-month follow up. Our analysis reveals that there was no significant difference in overall cost between the two groups 768.10 TL versus 758.04 TL (mean difference 10.07 TL, 95% BCa -861.28, 776.25) p=0.973). There was also no significant difference in any single element of costs to the health system or productivity losses.

**Appendix 7:** Unit costs (2020 Turkish Lira)

| **Type of Cost** | **Unit cost** | **Unit** | **Source and assumptions** |
| --- | --- | --- | --- |
| Community health worker (contact) | 39.77 | Per hour | Sourced from Pratisyen Hekim 15 Şubat Maaşları !Assume low scale point circa 7000 TL  [General practitioner salaries Feb 2020] https://www.saglikpersoneli.com.tr/gundem/pratisyen-hekim-15-subat-maaslari-h4938.html |
| Community-based doctor (contact) | 39.77 | Per hour | Sourced from Pratisyen Hekim 15 Şubat Maaşları !Assume low scale point circa 7000 TL  [General practitioner salaries Feb 2020] https://www.saglikpersoneli.com.tr/gundem/pratisyen-hekim-15-subat-maaslari-h4938.html |
| Psychiatrist (contact) | 13.00 | Per consultation | Sosyal Güvenlik Kurum [Social Security Institution] tariff for individual psychotherapy consultation |
| Psychologist (contact) | 13.00 | Per consultation | Sosyal Güvenlik Kurum [Social Security Institution] tariff for individual psychotherapy consultation |
| Psychiatric Nurse (contact) | 31.25 | Per hour | Range between 5,000TL and 6,000TL per month depending on experience; assume 5,500 TL per month |
| Social worker (contact) | 31.25 | Per hour | Range between 5,000TL and 6,000TL per month depending on experience; assume 5,500 TL per month |
| Psychiatric inpatient stay (nights) | 109.00 | Per night | Sosyal Güvenlik Kurum [Social Security Institution] tariff for inpatient stays for patients in category 2 of tariff code, including PTSD. F32.1 Moderate depressive episode; F33.1 Recurrent depressive disorder, current episode moderate; F34.0 Cyclothymia; (F40-48) Neurotic, stress-related and somatoform disorders (except for code groups F40, F45, F48); F50 Eating disorders; (F60-69) Adult personality and behavioral disorders; (F70-79) Mental retardation; F80 Speech and language specific developmental disorders; F84 Pervasive developmental disorders; F90 Hyperkinetic disorders; F91 Behavioural disorders; F92 Behavioural and emotional mixed type disorders |
| Other inpatient stay (nights) | 30.00 | Per night | Sosyal Güvenlik Kurum [Social Security Institution] tariff for standard non-mental health inpatient stays |
| Hospital Emergency Department (contact) | 15.00 | Per contact | Sosyal Güvenlik Kurum [Social Security Institution] tariff for standard non-mental health inpatient stays |
| Psychiatric outpatient (contact) | 13.00 | Per contact | Sosyal Güvenlik Kurum [Social Security Institution] tariff for individual psychotherapy consultation |
| Other outpatient (contact) | 13.00 | Per contact | Varies depending on speciality; we assume here same as SGK tariff for psychiatric outpatient contact |
| Day Hospital (Visit) | 6.00 | Per visit | Sosyal Güvenlik Kurum [Social Security Institution] tariff |
| Policlinic (Visit) | 15.00 | Per visit | Sosyal Güvenlik Kurum [Social Security Institution] tariff |
| Productivity Losses | 106 | Per day | Minimum Wage Determination Commission = 2324 TL per month; assume 22 working days per month = 106 TL per day |
|  |  |  |  |

| **Appendix 8:** Mean Difference in Cumulative Service Utilisation Per Participant at 3-Month Follow-Up (cases only) | | | | |
| --- | --- | --- | --- | --- |
| Type of Cost | PM+ | ETAU | Mean Difference (BCa 95% CI) | p |
| Health Service Utilisation, M (SD) | | | | |
| Community health worker (contact) | 0.08 (0.41) | 0.41 (0.85) | -0.33 (-0.71, 0.02) | 0.114 |
| Community-based doctor (contact) | 0.75 (1.82) | 1.09 (2.37) | -0.34 (-1.48, 0.76) | 0.590 |
| Psychiatrist (contact) | 0.42 (2.04) | 0.05 (0.21) | 0.37 (-0.11, 1.28) | 0.356 |
| Psychologist (contact) | 0.04 (0.20) | 0.00 | 0.04 (-0.04, 0.13) | 0.328 |
| Psychiatric Nurse (contact) | 0.00 | 0.00 | 0.00 | N/A |
| Social worker (contact) | 0.00 | 0.09 (0.43) | -0.09 (-0.28, 0.10) | 0.329 |
| Psychiatric inpatient stay (nights) | 0.00 | 0.09 (0.43) | -0.09 (-0.28, 0.10) | 0.329 |
| Other inpatient stay (nights) | 1.00 (4.06) | 1.55 (4.19) | -0.54 (-3.19, 2.03) | 0.657 |
| Hospital Emergency Department (contact) | 0.29 (0.91) | 0.77 (2.78) | -0.48(-1.56, 0.35) | 0.445 |
| Psychiatric outpatient (contact) | 0.00 | 0.05 (0.21) | -0.05 (-0.14, 0.05) | 0.329 |
| Other outpatient (contact) | 0.54 (1.91) | 0.14 (0.47) | 0.41 (-0.44, 1.25) | 0.323 |
| Day Hospital (Visit) | 0.42 (2.04) | 0.59 (2.56) | -0.17 (-1.58, 1.10) | 0.801 |
| Policlinic (Visit) | 0.46 (1.22) | 0.82 (2.02) | -0.36 (-1.40, 0.49) | 0.473 |
| Medicine (doses) | 7.58 (37.15) | 12.41 (58.20) | -4.83 (-34.11, 21.84) | 0.766 |
| CAM (contact) | 0.00 | 0.14 (0.64) | 0.14 (-0.42, 0.15) | 0.329 |
| Productivity Loss (days) | 6.58 (9.33) | 15.86 (59.42) | -9.28 (-32.41, 6.36) | 0.499 |

*Cumulative service utilisation combining post assessment and 3-month follow up periods

*Mean (SD) at baseline, post-assessment and three month follow up in costs (Turkish Lira). (complete cases only – no imputed data)*

| Service | **Baseline** | | **Post-assessment** | | **3 MFU** | |
| --- | --- | --- | --- | --- | --- | --- |
|  | PM+ (n=24) | ETAU (n=22) | PM+ (n=24) | ETAU n=21) | PM+ (n=20) | ETAU (n=20) |
| Community health worker (contact) | 0.41 (2.03) | 9.61 (35.57) | 0.00 | 0.30 (1.41) | 0.83 (4.06) | 1.21 (2.62) |
| Community-based doctor (contact) | 11.19 (6.39) | 6.39 (11.26) | 1.80 (6.98) | 1.51 (7.07) | 6.35 (20.43) | 25.91 (73.89) |
| Psychiatrist (contact) | 0.00 | 11.81 (55.43) | 0.00 | 0.59 (2.77) | 5.42 (26.54) | 0.00 |
| Psychologist (contact) | 0.00 | 0.00 | 0.00 | 0.00 | 0.54 (2.65) | 0.00 |
| Psychiatric Nurse (contact) | 0.00 | 0.00 | 0.00 | 0.00 | 0.00 | 0.00 |
| Social worker (contact) | 0.00 | 0.28 (1.33) | 0.00 | 0.00 | 0.00 | 1.66 (7.77) |
| Psychiatric inpatient stay (nights) | 0.00 | 0.00 | 4.54 (22.25) | 0.00 | 0.00 | 9.91 (46.48) |
| Other inpatient stay (nights) | 7.50 (36.74) | 1.36 (6.40) | 27.50 (122.23) | 39.55 (120.85) | 2.50 (8.47) | 6.82 (15.85) |
| Hospital Emergency Department (contact) | 3.75 (11.06) | 8.18 (31.98) | 4.38 (13.62) | 0.00 | 0.00 | 11.59 (41.64) |
| Psychiatric outpatient (contact) | 0.00 | 0.00 | 0.00 | 0.59 (2.77) | 0.00 | 0.00 |
| Other outpatient (contact) | 16.79 (35.65)* | 1.18 (5.54)* | 0.00 | 0.00 | 7.04 (24.84) | 1.77 (6.08) |
| Day Hospital (Visit) | 0.00 | 4.09 (11.17) | 2.50 (12.25) | 0.00 | 0.00 | 3.55 (15.34) |
| Policlinic (Visit) | 4.38 (16.24) | 5.45 (22.46) | 4.38 (16.24) | 5.45 (22.46) | 2.50 (9.55) | 6.82 (22.07) |
| Medicine (doses) | 0.00 | 0.00 | 0.00 | 0.00 | 0.00 | 0.00 |
| CAM (contact) | 26.67 (90.39) | 16.36 (64.63) | 0.00 | 0.00 | 0.00 | 0.00 |
| Productivity Loss (days) | 923.08 (2014.24) | 269.82 (712.00) | 640.42 (937.98) | 269.82 (557.77) | 57.42 (153.06) | 371.00 (1099.52) |

* p<0.05

Able to collect CSRI data on contacts with services; low use of services for most individuals; no significant differences between utilisation rates at each time point for all categories (using parametric test) other than for other outpatient contacts at baseline.

| **Appendix 9:** Mean Difference in Cumulative Costs Per Participant at 3-Month Follow-Up (cases only) | | | | |
| --- | --- | --- | --- | --- |
| **Type of Cost** | **PM+** | **ETAU** | **Mean Difference (BCa 95% CI)** | **p** |
| Cost (Turkish Lira), M (SD) | | | | |
| Community health worker (contact) | 0.83 (4.06) | 1.51 (3.19) | -0.68 (-2.84, 1.48) | 0.530 |
| Community-based doctor (contact) | 8.15 (22.35) | 27.42 (74.38) | -19.27 (-55.58, 6.49) | 0.292 |
| Psychiatrist (contact) | 5.42 (26.54) | 0.59 (2.77) | 4.83 (-1.63, 17.18) | 0.334 |
| Psychologist (contact) | 0.54 (2.65) | 0.00 | 0.54 (-0.58, 1.66) | 0.135 |
| Psychiatric Nurse (contact) | 0.00 | 0.00 | 0.00 | N/A |
| Social worker (contact) | 0.00 | 1.65 (7.77) | -1.66 (-5.83, 1.79) | 0.107 |
| Psychiatric inpatient stay (nights) | 4.54 (22.25) | 9.91 (46.48) | -5.36 (-29.72, 11.86) | 0.626 |
| Other inpatient stay (nights) | 30.00 (121.94) | 46.36 (125.83) | -16.36 (-91.22, 52.00) | 0.657 |
| Hospital Emergency Department (contact) | 4.38 (13.62) | 11.59 (41.64) | -7.22 (-28.00, 6.57) | 0.445 |
| Psychiatric outpatient (contact) | 0.00 | 0.59 (2.77) | -0.59 (-1.82, 0.64) | 0.118 |
| Other outpatient (contact) | 7.04 (24.84) | 1.77 (6.08) | 5.27 (-1.60, 14.77) | 0.323 |
| Day Hospital (Visit) | 2.50 (12.25) | 3.55 (15.34) | -1.05 (-10,63, 7.36) | 0.801 |
| Policlinic (Visit) | 6.88 (18.23) | 12.27 (30.23) | -5.40 (-21.53, 9.80) | 0.473 |
| Medicine (doses) | 0.00 | 0.00 | 0.00 | N/A |
| CAM (contact) | 0.00 | 0.00 | 0.00 | N/A |
| Productivity Loss (days) | 697.83 (989.40) | 640.82 (1565.23) | 57.02 (-784.42, 791.45) | 0.885 |
| **Total cost** | **768.10 (1009.66)** | **758.04 (1664.18)** | **10.07 (-861.28, 776.25)** | **0.973** |

* Mean difference in cumulative costs combining post assessment and 3-month follow up periods

**Appendix 10:** Fidelity Checklist

**SESSION 1**

| No. | Item | Components Checklist  (Circle or mark each completed) | Competency Scoring  (Circle one) |
| --- | --- | --- | --- |
| 1.1 | Conduct **Opening Procedures** | 1.1a – Welcome and introductions (Give name cards to write)  1.1 b – Logistics, session and role information  1.1 c - Hang group guideline poster and discuss with participants | 1 Needs Improvement = *does not complete all tasks*  2 Done Partially = *completes tasks but does not facilitate discussion on tasks (i.e., didactic only)*  3 Done Well = *completes all tasks and facilitates discussion on tasks (e.g., interactive with participants, provides opportunity for questions, answers questions)* |
| 1.2 | Explain and Promote **Confidentiality** | 1.2a – Explain concept of confidentiality in group treatment  1.2b – Provide information about when confidentiality can be broken | 1 Needs Improvement = *does not complete all tasks*  2 Done Partially = *tells participants what confidentiality is and when it can be broken, but does not give reason why and does not remind participants at end*  3 Done Well = *explains confidentiality and when it can be broken, explains why, and reminds participants at end of session* |
| 1.3 | Introduce **What is PM+?** | 1.3a – Hang ‘What is PM+?’ and ‘4 Strategies’ posters and discuss  1.3b – Read case examples 1,2,3, and 4 and discuss | 1 Needs Improvement = *does not complete all tasks*  2 Done Partially = *completes tasks but does not facilitate discussion on case examples (i.e., didactic only)*  3 Done Well = *completes all tasks and facilitates discussion on tasks (e.g., interactive with participants about case examples, provides opportunity for questions, answers questions)* |
| 1.4 | Set **Collaborative Goals** with Participants | 1.4a – Discuss participant’s reasons and challenges for attending group  1.4b –Discussion on individual goals  1.4 c – Read case examples 5 and 6 and discuss | 1 Needs Improvement = *does not complete all tasks*  2 Done Partially = *completes tasks but does not facilitate discussion on tasks (i.e., didactic only)*  3 Done Well = *completes all tasks and facilitates discussion on tasks (e.g., interactive with participants about goals and case examples, provides opportunity for questions, answers questions)* |
| 1.5 | Conduct **What is Adversity** | 1.5a – Define adversity (refer to Activity – 4 Posters of Adversity)  1.5 b – Discussion on common reactions to adversity | 1 Needs Improvement = *does not complete all tasks*  2 Done Partially = *completes tasks but does not facilitate discussion on tasks (i.e., didactic only)*  3 Done Well = *completes all tasks and facilitates discussion on tasks (e.g., interactive with participants about concept of adversity in their own lives, provides opportunity for questions, answers questions)* |
| 1.6 | Teach and Practice **Managing Stress Exercise** | 1.6a – Provide information on how stress affects the body  1.6 b – Complete breathing activity (counting breaths)  1.6 c – Teach and practice breathing from the diaphragm/stomach (show balloon)  1.6 d – Practice slow breathing together  1.6 e – Discuss challenges and difficulties  1.6 f – Read case example 7 and discuss | 1 Needs Improvement = *does not complete all tasks*  2 Done Partially = *completes tasks but does not work to ensure each participant can complete skill*  3 Done Well = *completes all tasks and ensures each participant can complete skill* |
| 1.7 | Uses appropriate **psychosocial communication skills** | 1.8a – Appropriate eye contact, facial expression, and body language  1.8b – Demonstrates a non-judgmental attitude  1.8c – Appropriate use of non-verbal communication  1.8d – Communicates concern and validates participants | 1 Needs Improvement = *shows judgement, gives advice to participants, fails to manage problem group dynamics and fails to use appropriate psychosocial communication skills*  2 Done Partially = *partial use of appropriate psychosocial communication skills but not throughout session*  3 Done Well = *consistently uses appropriate psychosocial communication skills* |
| 1.8 | Incorporates **safety management skills** | 1.8a –Reviews for suicidality if necessary  1.8 b - Identifies potentials risks of harm to self or others  1.8c – Uses techniques for acute management of risk and provides referral | 1 Needs Improvement = *does not identify potential harm;*  2 Done Partially = *identifies potential harm but provides incomplete response*  3 Done Well = *identifies potential harm and implements comprehensive safety plan*  4 Not applicable |
| 1.9 | Appropriately uses **group facilitation skills** | 1.9a – Encourages all participants to participate  1.9b – Prevents or manages dominant participants  1.9c – Encourages group members to propose ideas and solutions for one another and prevents criticism of one another  1.9d – Redirects group back to themes of session if they talk off-topic | 1 Needs Improvement = *lets one or two people dominate entire session; allows member to speak negatively or critically of one another*  2 Done Partially = *all group members participate, but they do not interact with and support one another*  3 Done Well = *all group members participate and share suggestions and supportive comments with one another* |
| 1.10 | **Closing Procedures** | 1.10a - Provide information for referral and connection with other services (at the end of session)  1.10b – Review of session and Schedule home practice  1.10 c- Information of next session (remind date, time, place and strategy) | 1 Needs Improvement = *does not complete all tasks*  2 Done Partially = *completes tasks but does not check participants are confident completing tasks*  3 Done Well = *completes all tasks and ensures participants are confident completing tasks* |

**SESSION 2**

| No. | Item | Components (Circle all that apply) | Scoring (circle one) |
| --- | --- | --- | --- |
| 2.1 | Incorporates **safety management skills** | 2.1a –Reviews for suicidality if necessary  2.1 b - Identifies potentials risks of harm to self or others  2.1c – Uses techniques for acute management of risk and provides referral | 1 Needs Improvement = *does not identify potential harm;*  2 Done Partially = *identifies potential harm but provides incomplete response*  3 Done Well = *identifies potential harm and implements comprehensive safety plan*  4 Not applicable |
| 2.2 | Conduct **Opening Procedures** | 2.3a – Welcome the group and share session plan  2.3b – Remind about group guideline | 1 Needs Improvement = *does not complete all tasks*  2 Done Partially = *completes tasks but does not facilitate discussion on tasks (i.e., didactic only)*  3 Done Well = *completes all tasks and facilitates discussion on tasks (e.g., interactive with participants, provides opportunity for questions, answers questions)* |
| 2.3 | Promote **Confidentiality** | 2.2a – Remind participants about importance of confidentiality at intro and conclusion of session  2.2b – Respond to potential violations of confidentiality or need to break confidentiality | 1 Needs Improvement = *does not complete all tasks*  2 Done Partially = *reminds participants about confidentiality*  3 Done Well = *reminds participants about confidentiality at beginning and end, and responds to potential breaches* |
| 2.4 | Review **Managing Stress** | 2.4 a – Read case example 8  2.4 b - Review Participants’ Managing stress homepractice  2.4c – Help manage any difficulties with home practice | 1 Needs Improvement = *does not complete all tasks*  2 Done Partially = *completes tasks but does not facilitate discussion on tasks (i.e., didactic only) or supports participants who had difficulties*  3 Done Well = *completes all tasks and facilitates discussion on tasks (e.g., interactive with participants, provides opportunity for questions, answers questions) and supports participants who had difficulties* |
| 2.5 | Introduce **Managing Problems** | 2.5a – Introduce strategy Managing problems for practical problems  2.5b – Discuss the differences between solvable and unsolvable problems (refer to Activity-Participants in the line)  2.5c – Hang Managing Problems chart and read 7 steps  2.5d – Read case example 9, 10, 11, 12, 13 and 14 | 1 Needs Improvement = *does not complete all tasks*  2 Done Partially = *completes tasks but does not facilitate discussion on case examples (i.e., didactic only)*  3 Done Well = *completes all tasks and facilitates discussion on tasks (e.g., interactive with participants about case examples, provides opportunity for questions, answers questions)* |
| 2.6 | Managing problems activities with participants | 2.6a – Ask all participants to think their problems and chose one  2.6b – Prioritize and chose a problem from participants (e.g. difficulty finding work)  2.6c – Ask the participant to define the problem  2.6d – Ask them what is the next step  2.6e – Let the participant brainstorms potential solutions for him/herself, and other participants brainstorm potential solutions  2.6f – Ask the participant to decide and chose one or some solutions  2.6g – Help him/her to develop action plan  2.6h – Small groups complete personal Managing Problems steps  2.6h – Review at least several participants’ action plans for managing problems | 1 Needs Improvement = *does not complete all tasks*  2 Done Partially = *completes tasks but does not facilitate discussion on tasks (i.e., didactic only) or work to ensure each participant can complete skill*  3 Done Well = *completes all tasks and facilitates discussion on tasks (e.g., interactive with participants about goals and case examples, provides opportunity for questions, answers questions), and ensures each participant can complete skills* |
| 2.7 | Practice **Managing Stress** | 2.7a – Review breathing from the stomach  2.7b – Practice slow breathing together  2.7c – Discuss challenges and difficulties | 1 Needs Improvement = *does not complete all tasks*  2 Done Partially = *completes tasks but does not work to ensure each participant can complete skill*  3 Done Well = *completes all tasks and ensures each participant can complete skill* |
| 2.8 | Uses appropriate **psychosocial communication skills** | 2.8a – Appropriate eye contact, facial expression, and body language  2.8b – Demonstrates a non-judgmental attitude  2.8c – Appropriate use of non-verbal communication  2.8d – Communicates concern and validates participants | 1 Needs Improvement = *shows judgement, gives advice to participants, fails to manage problem group dynamics and fails to use appropriate psychosocial communication skills*  2 Done Partially = *partial use of appropriate psychosocial communication skills but not throughout session*  3 Done Well = *consistently uses appropriate psychosocial communication skills* |
| 2.9 | Appropriately uses **group facilitation skills** | 2.9a – Encourages all participants to participate  2.9b – Prevents or manages dominant participants  2.9c – Encourages group members to propose ideas and solutions for one another and prevents criticism of one another  2.9d – Redirects group back to themes of session if they talk off-topic | 1 Needs Improvement = *lets one or two people dominate entire session; allows member to speak negatively or critically of one another*  2 Done Partially = *all group members participate, but they do not interact with and support one another*  3 Done Well = *all group members participate and share suggestions and supportive comments with one another* |
| 2.10 | **Closing Procedures** | 2.10a - Provide information for referral and connection with other services (at the end of session)  2.10b – Review of session and Schedule home practice  2.10 c- Information of next session (remind date, time, place and strategy) | 1 Needs Improvement = *does not complete all tasks*  2 Done Partially = *completes tasks but does check participants are confident completing tasks*  3 Done Well = *completes all tasks and ensures participants are confident completing tasks* |
